# Tumor biomarker conversion between primary and metastatic breast cancer: mRNA assessment and its concordance with immunohistochemistry

**DOI:** 10.18632/oncotarget.18006

**Published:** 2017-05-19

**Authors:** Stefan Stefanovic, Ralph Wirtz, Thomas M. Deutsch, Andreas Hartkopf, Peter Sinn, Zsuzsanna Varga, Bettina Sobottka, Lakis Sotiris, Florin-Andrei Taran, Christoph Domschke, Andre Hennigs, Sara Y. Brucker, Christof Sohn, Florian Schuetz, Andreas Schneeweiss, Markus Wallwiener

**Affiliations:** ^1^ Department of Gynecology and Obstetrics, Heidelberg University Hospital, 69120 Heidelberg, Germany; ^2^ National Center for Tumor Diseases (NCT) Heidelberg, 69120 Heidelberg, Germany; ^3^ Stratifyer Molecular Pathology GmbH, 50935 Cologne, Germany; ^4^ Department of Women's Health, University Hospital Tübingen, 72076 Tübingen, Germany; ^5^ Department of Pathology, Heidelberg University Hospital, 69120 Heidelberg, Germany; ^6^ Institute of Surgical Pathology, Zurich University Hospital, 8091 Zurich, Switzerland; ^7^ Research Institute for Women's Health, Tübingen University Hospital, 72076 Tübingen, Germany

**Keywords:** breast cancer, tumor biomarkers, receptor conversion, immunohistochemistry, real-time quantitative polymerase chain reaction

## Abstract

Biomarker changes between primary (PT) and metastatic tumor (MT) site may be significant in individualizing treatment strategies and can result from actual clonal evolution, biomarker conversion, or technical limitations of diagnostic tests.

This study explored biomarker conversion during breast cancer (BC) progression in 67 patients with different tumor subtypes and metastatic sites via mRNA quantification and subsequently analyzed the concordance between real-time qPCR and immunohistochemistry (IHC). Immunostaining for estrogen receptor (ER), progesterone receptor (PR), HER2, and Ki-67 was performed on formalin-fixed, paraffin-embedded PT and MT tissue sections. RT-qPCR was performed using a multiplex RT-qPCR kit for *ESR1*, *PGR*, *ERBB2*, and *MKI67* and the reference genes *B2M* and *CALM2*.

Subsequent measurement of tumor biomarker mRNA expression to detect conversion revealed significant decreases in *ESR1* and *PGR* mRNA and *MKI67* upregulation (all *p* < 0.001) in MT compared to PT of all tumor subtypes and *ERBB2* upregulation in MT from triple-negative PT patients (*p* = 0.023). Furthermore, *ERBB2* mRNA was upregulated in MT brain biopsies, particularly those from triple-negative PTs (*p* = 0.023). High concordance between RT-qPCR and IHC was observed for ER/*ESR1* (81%(κ 0.51) in PT and 84%(κ 0.34) in MT, PR/*PGR* (70%(κ 0.10) in PT and 78% (κ −0.32) in MT), and for HER2/*ERBB2* (100% in PT and 89% in MT). Discordance between mRNA biomarker assessments of PT and MT resulting from receptor conversion calls for dynamic monitoring of BC tumor biomarkers. Overall, RT-qPCR assessment of BC target genes and their mRNA expression is highly concordant with IHC protein analysis in both primary and metastatic tumor.

## INTRODUCTION

Tremendous progress has been made in the treatment of metastatic breast cancer (MBC) in recent decades, but still very few therapies use patient or tumor-specific characteristics to tailor individualized treatment [1, 2]. Breast cancer (BC) is a disease with heterogeneous tumor biology, comprising divergent molecular subtypes [3–5]. In this context, one of the main challenges is to minimize overtreatment by developing precise patient selection criteria for targeted therapies by defining new biomarkers or real-time monitoring of tumor biology dynamics and minimal residual disease (MRD) [1, 6].

Clinical decision making in both primary and MBC is based on pathologic assessment of tumor biomarkers, preferably target genes or proteins acquired from primary tumor biopsy.

There is a growing body of evidence describing tumor biomarker conversion, i.e. upregulation or downregulation of hormone receptors, HER2/*ERBB2* and Ki-67/*MKI67* when comparing metastatic tumors (MT) with primary tumors (PT) [7–19]. The ability to capture tumor biomarker changes in the course of BC progression could have consequences for further diagnostic procedures, in the context of liquid biopsies [20, 21] or conventional rebiopsies [22, 23], as well as potential therapeutic implications, especially for trials aiming for an extended indication of HER2 targeted therapies. Conventional rebiopsies upon reaching the next stage of cancer progression are a suboptimal method due to their limited feasibility and invasiveness.

Identifying associations between immunophenotype and patterns of the metastatic spread of BC could help define surrogate markers to identify patients with a high risk for developing cerebral metastases based on tumor biomarker status at the time of primary diagnosis. Therefore, reliable and sensitive methods of quantifying changes in target tumor biomarker expression in the course of cancer progression are of utmost importance for optimizing treatment strategies in the metastatic situation.

Apart from substantial biomarker conversion at the mRNA/protein level or even clonal evolution as genetic alterations – mainly hotspot mutations [24–29], another potential scenario behind the conversion of tumor biomarker is due to technical limitations of dichotomous biomarker assessment via conventional IHC. Exact quantification of the underlying target gene and protein expression could essentially facilitate exploration of tumor biomarker conversion upon disease progression, and therefore the present study used a highly standardized real-time quantitative polymerase chain reaction (RT-qPCR) kit to compare the expression levels of the *ESR1, PGR, ERBB2*, and *MKI67* genes in matched pairs of BC PT and MT samples. In addition, matched results from conventional immunohistochemistry assessments of the same biomarkers were compared to address the concordance or discordance between the mRNA and protein assessments.

The study objective was to use mRNA quantification to explore biomarker conversion in different tumor subtypes and metastatic locations during breast cancer progression and to analyze the concordance between RT-qPCR and IHC in PT and MT samples.

## RESULTS

Matched BC PT and MT samples were available from 67 patients. Median age at the time of primary BC diagnosis was 51.9 years. Median age at first diagnosis of metastatic disease was 56.9 years. As shown in Table 1, patients with the luminal subtype (hormone receptor positive, HER2 negative as assessed by IHC of the PT) comprised 62% of the total cohort.

**Table 1:** Clinicopathological patient characteristics

Patient characteristic		*n* (%)
Total, *n*		67
Median age at PT biopsy, years		51,9
Median age at MT biopsy, years		56,9
Phenotype of primary tumor by IHC, n (%)		
Luminal A		25 (37%)
Luminal B (HER2-negative)		15 (22%)
Luminal B (HER2-positive)		2 (3%)
HER2 positive (non-luminal)		6 (9%)
Triple-negative		17 (25%)
NA		2 (3%)
Grading of primary tumor, *n* (%)		
G1		
G1		0 (0%)
G2		40 (60%)
G3		20 (30%)
GX		7 (10%)
ER/*ESR1* status of primary tumor, *n* (%)	**IHC**	**RTqPCR**
Positive	44 (66%)	42 (63%)
Negative	23 (34%)	24 (36%)
Unknown	0 (0%)	1 (2%)
PR/*PGR* status of primary tumor, *n* (%)		
Positive	42 (63%)	29 (43%)
Negative	25 (37%)	37 (55%)
Unknown	0 (0%)	1 (2%)
HER2/*ERBB2* status of primary tumor, *n* (%)		
Positive	14 (21%)	8 (12%)
Negative	39 (58%)	58 (87%)
Unknown	14 (21%)	1 (2%)
Site of metastatic biopsy, *n* (%)		
Bone		24 (36%)
Brain		19 (28%
Liver		8 (12%)
Lung		2 (3%)
Pleura		3 (5%)
Soft tissue		11 (16%)

**Figure 1:**
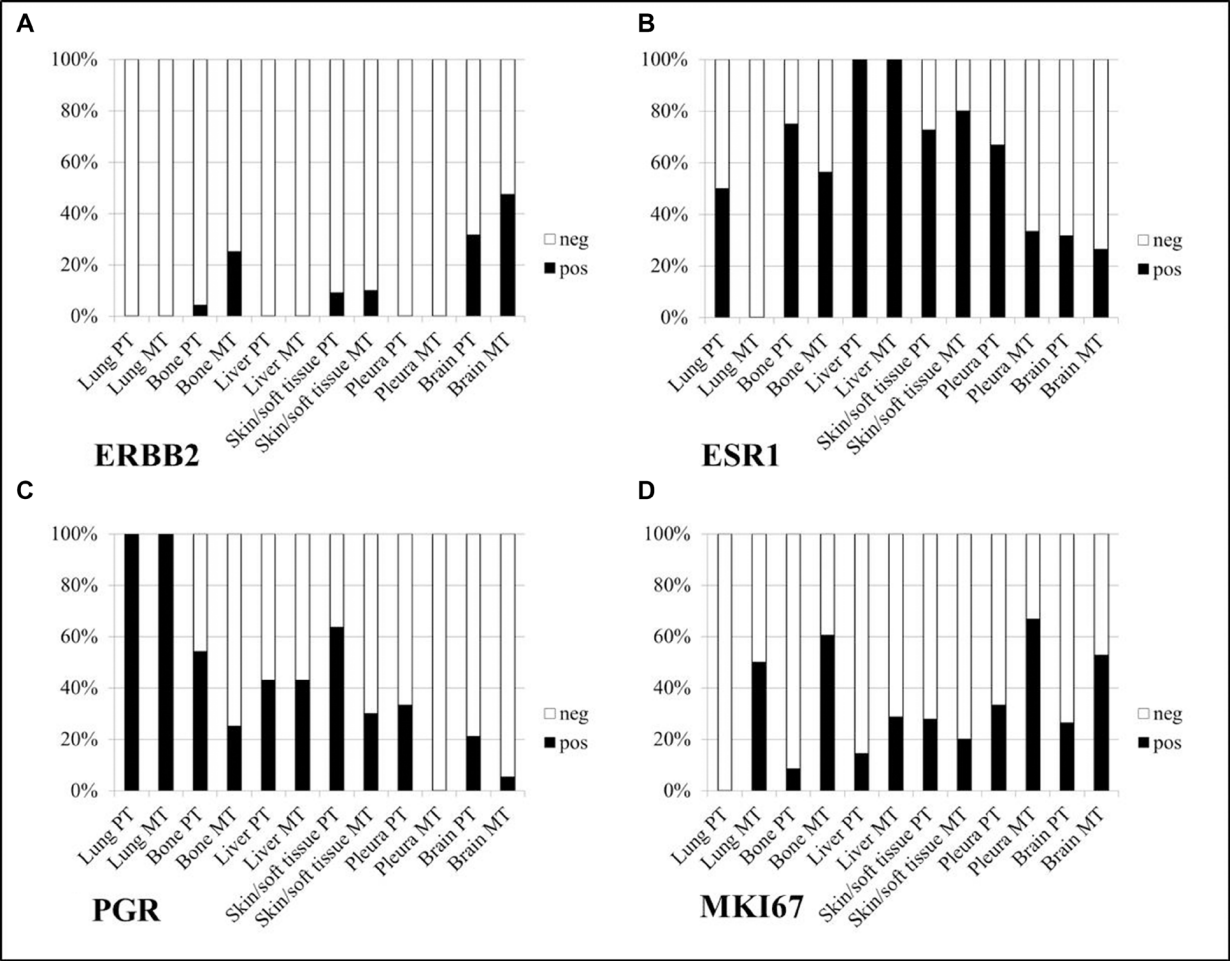
mRNA assessment of tumor biomarkers in metastatic tumor (MT) vs. primary (PT) tumor samples, by biopsy site

This study initially compared the dynamics of tumor biomarkers in PTs vs. MTs by biopsy site using RT-qPCR. The mRNA assessment showed *ESR1* downregulation in MT (compared to PT) samples from all biopsy sites, the difference being statistically significant in the brain (*40−ΔΔCT* *p <* 0.001). *PGR* was downregulated in MT (vs. PT) from all biopsy sites, the difference being significant in the brain (*40−ΔΔCT p <* 0.001), liver (*40−Δ*Δ*CT* *p* = 0.004), and skin/soft tissue samples (*40−ΔΔCT* *p* = 0.006). *MKI67* was upregulated in MT (vs. PT) in samples from all biopsy sites, the difference being significant in the brain (*40−ΔΔCT* *p* = 0.034), bone (*40−ΔΔCT* *p <* 0.001), and skin/soft tissue (*40−ΔΔCT* *p* = 0.018). Furthermore, a modest *ERBB2* upregulation trend in MT (vs. PT) was recorded in patients with MT in biopsies from the brain (*40−ΔΔCT* *p* = 0.138) and bone (*40−ΔΔCT* *p* = 0.138), see Figure 1.

**Figure 2:**
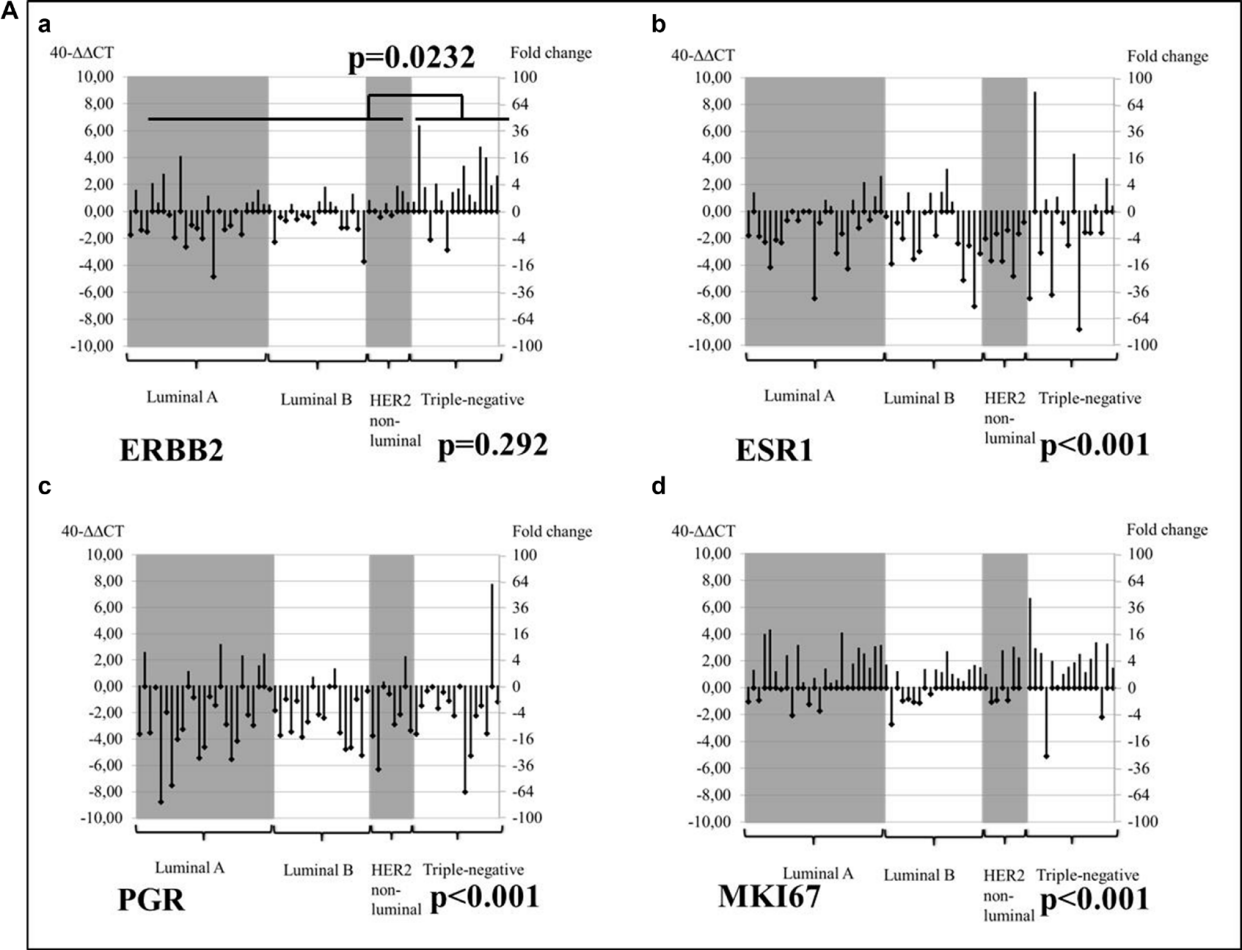

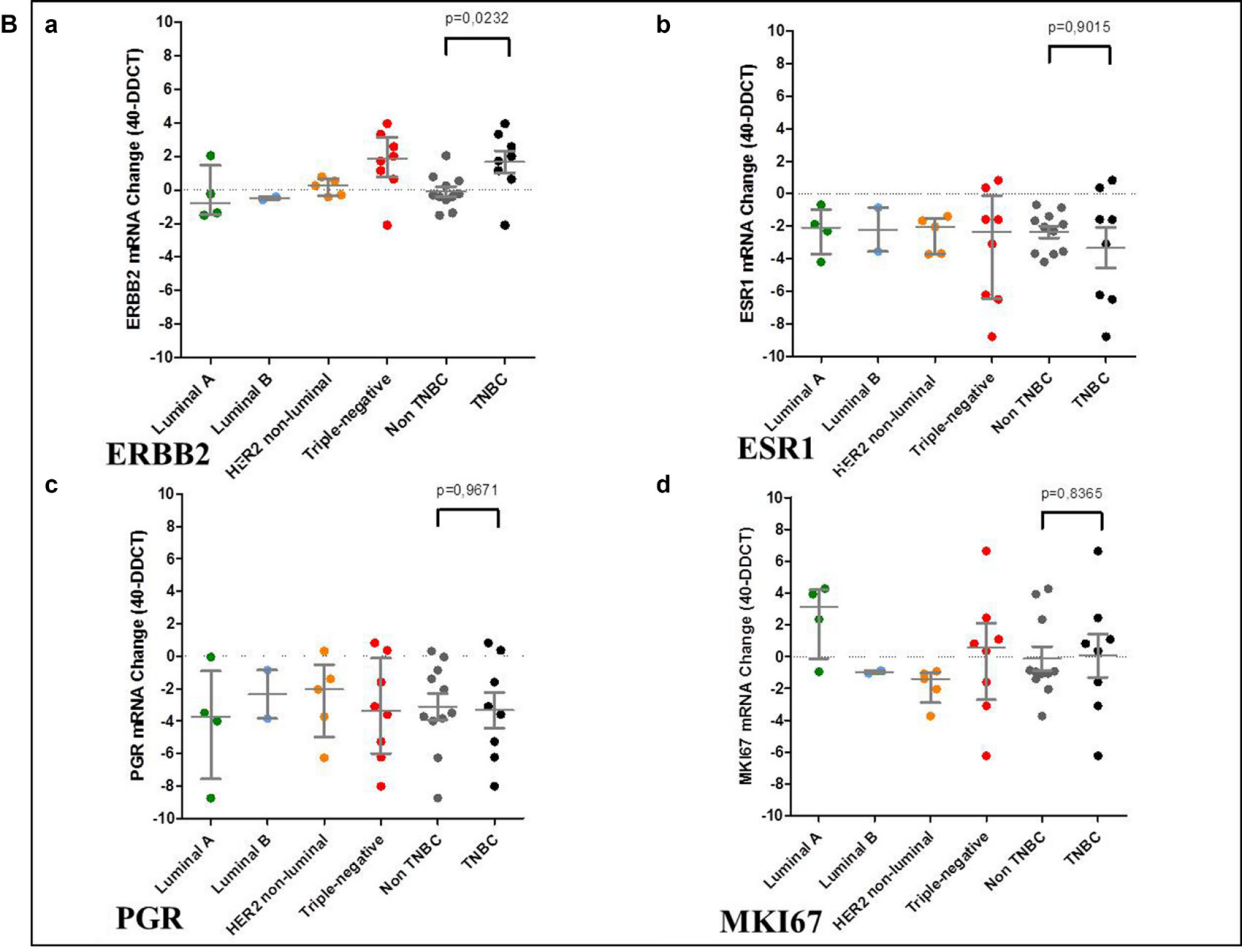
Changes from PT baseline in the MT mRNA levels of tumor biomarkers, by tumor subtype (**A**) 40−ΔΔ Cq; (**B**) Mann–Whitney test.

Subsequently, changes in tumor biomarker mRNA expression were measured in MT compared to the PT baseline for each phenotype. Significant decreases in *ESR1* and *PGR* mRNA and upregulation of *MKI67* (all *p <* 0.001) were observed in MT vs. PT of all phenotypes (Figure 2A). *ERBB2* upregulation was significant in MT from triple-negative vs. non-triple-negative PT patients (*p* = 0.023, Mann–Whitney U Test), as shown in Figure 2B. An adjacent analysis focused on the dynamics of tumor biomarker mRNA expression by tumor subtype in MT vs. PT samples from particular metastatic sites.

### Brain metastasis

**Figure 3:**
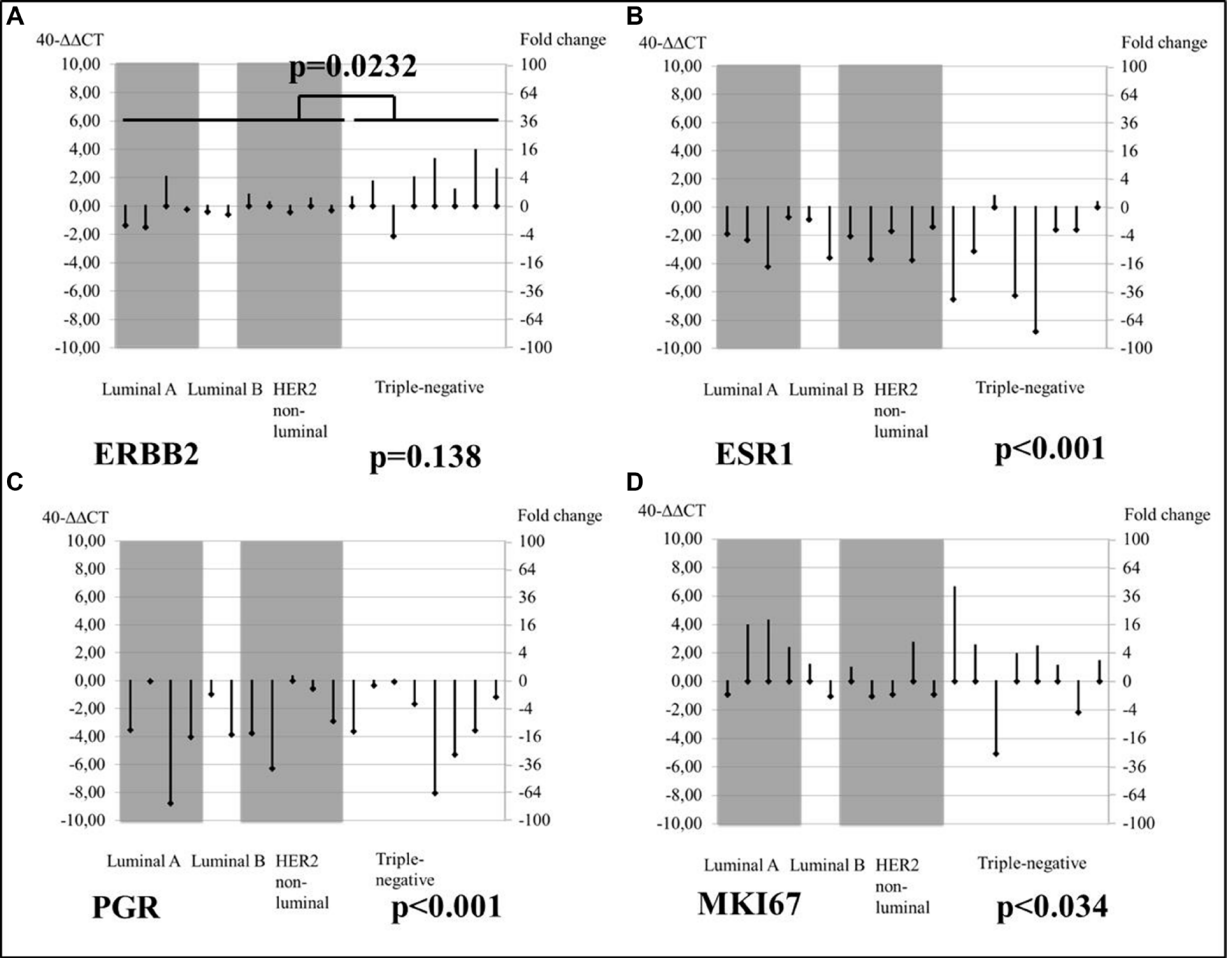
Changes from PT baseline in brain MT mRNA levels of tumor biomarkers, by tumor subtype

*ESR1/PGR* expression was significantly downregulated (*p <* 0.001) in brain MT biopsies for all tumor subtypes (compared to PT), while *ERBB2* mRNA was upregulated in brain MT biopsies, particularly in triple-negative PTs (*p* = 0.023), as shown in Figure 3.

### Bone metastasis

**Figure 4:**
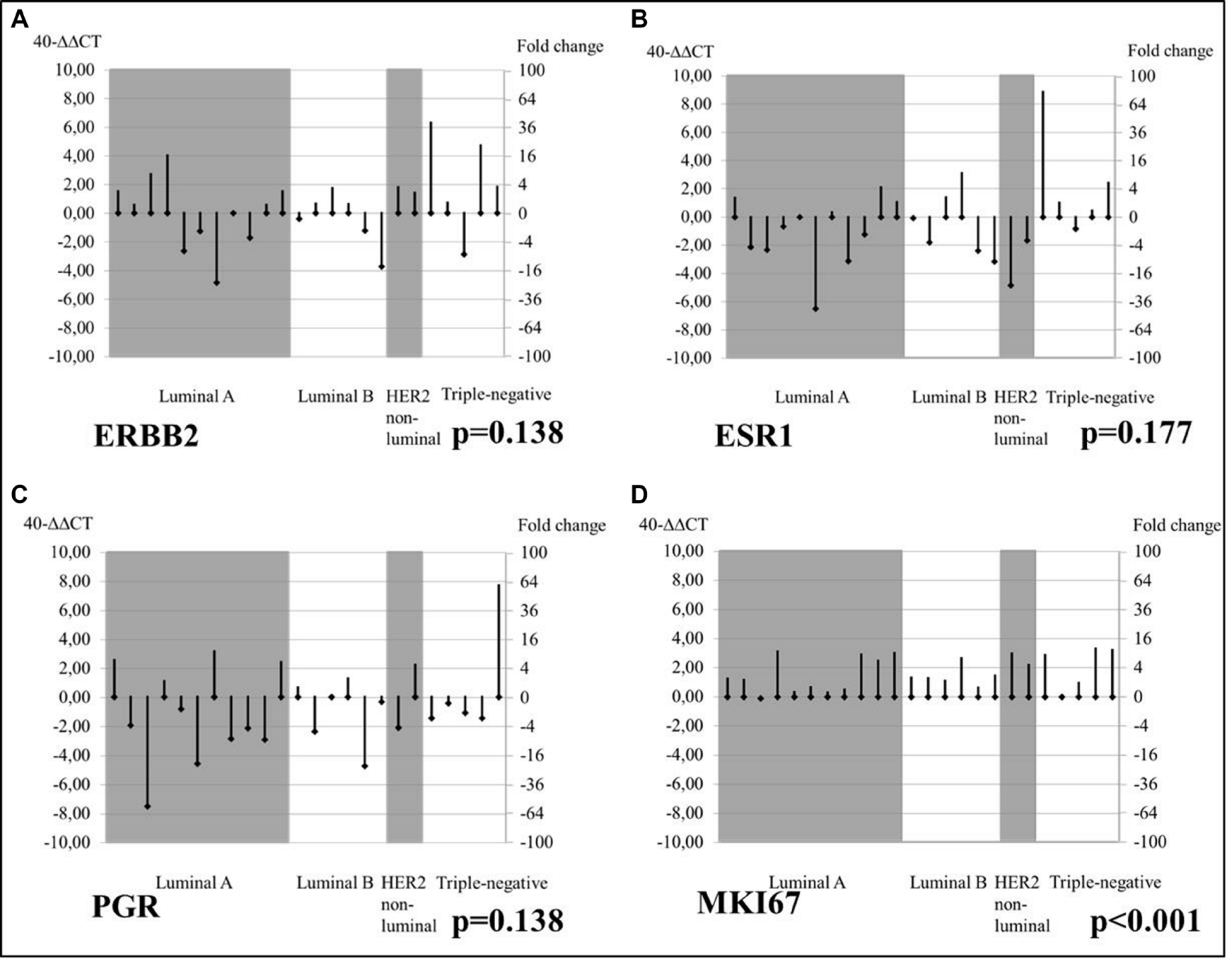
Changes from PT baseline in bone MT mRNA levels of tumor biomarkers, by tumor subtype

Accordingly, the study assessed the conversion of tumor biomarkers in MT vs. PT baseline by immunophenotype in bone-acquired biopsies using RT-qPCR. *ESR1* and *PGR* expression levels were modestly downregulated in bone MT compared to PT biopsies for all immunophenotypes (*p* = 0.177 and *p* = 0.138, respectively), while *ERBB2* mRNA was upregulated in bone MT biopsies, particularly in triple-negative PTs (*p* = 0.138), as shown in Figure 4.

### Concordance

For each biomarker, we calculated the concordance between the RT-qPCR result and the hormone and HER2 status as established by IHC and obtained from diagnostic pathology reports. High concordance between RT-qPCR and IHC assessments was observed for ER/*ESR1*: 81% (κ 0.51) in PT and 84% (κ 0.34) in MT; PR/*PGR*: 70% (κ 0.10) in PT and 78% (κ −0.32) in MT; and HER2/*ERBB2*: 100% in PT and 89% in MT, see Table 2. As regards the recognition of discordance in receptor conversion, three patients showed a change from ER-negative PT to ER-positive MT as assessed by RT-qPCR. These biopsies were obtained from one lung MT and two bone MTs. IHC showed that in four patients a “non-luminal” PT converted to a “luminal” MT exhibiting hormone receptor expression, while only one such case was observed with RT-qPCR.

**Table 2:** (A) Concordance calculation between RT-qPCR and IHC for the biomarkers studied, (B) Methodologic discrepancies in receptor conversion recognition

**A) Concordance of RT-qPCR and IHC**	**ER/ESR1**	**PR/PGR**
Primary breast cancer (PT)	81% κ 0.51	70% κ 0.10
Metastatic breast cancer (MT)	84% κ 0.34	78% κ−0.32
**B) Hormone receptor conversion**	**RT-qPCR**	**IHC**
Total:	19 (20)*/55°	21 (22)*/53†
Both neg *to* both pos	0	1
Both pos *to* both neg	4	4
ER neg *to* pos/PR neg	1	1
ER neg *to* pos/PR pos	1	3
ER pos *to* neg/PR neg	2	0
ER pos *to* neg/PR pos	1	1
PR neg *to* pos/ER neg	(1)*	1
PR neg *to* pos/ER pos	0	1
PR pos *to* neg/ER neg	0	1
PR pos *to* neg/ER pos	8	8 (9)*

*One patient had 2 MT biopsies for one PT, but only one MT biopsy showed a shift, thus the value including the shifting sample in brackets.

°RT-qPCR total comprises 55 patients as % tumor cells were insufficient for accurate statement of hormone receptor positivity/negativity in 11 MT and 1 PT.

†IHC total comprises 53 samples as immunohistochemistry was missing for 14 samples.

## DISCUSSION

### Biomarker conversion: ERBB2 overexpression in initially HER2 negative metastatic breast cancer

Recently, a growing body of evidence has emerged, confirming that a shift in hormone receptor status from positive in PT to negative in MT is associated with worse survival compared with consistent endocrine sensitivity. Hormone receptors have been shown to be independent prognostic factors for post-recurrence BC mortality in multivariate analyses at all stages of tumor progression [23, 30–32]. Our analysis was able to confirm a tendency in biomarker conversion towards a more aggressive phenotype with significant downregulation of *ESR1* and *PGR* in biopsies from all MT sites and in all molecular subtypes of MT, as well as a boost in the proliferation marker *MKI67* in all phenotypes (*p <* 0.001).

Apart from the expected downregulation of hormone receptors and upregulation of Ki-67 in all tumor subtypes of MT compared to PT, *ERBB2* upregulation was observed in MT samples from triple-negative PT patients (*p* = 0.292), whereas *ERBB2* overexpression was particularly significant in brain MTs in initially triple-negative PT BC (*p* = 0.023). Other trials comparing HER2 PT and MT status have evidenced HER2 receptor dynamics [22, 33–36], but the present study is one of the first to demonstrate significantly increased HER2 levels in cerebral entities originating from triple-negative BC PTs [37–40].

### Diagnostic potential for liquid biopsy and rebiopsy, and the therapeutic consequences

The discordances between PT and MT mRNA biomarker assessments caused by receptor conversion call for dynamic monitoring of BC tumor biomarkers, preferably via liquid biopsy or via rebiopsy of metastatic lesions, based on soluble receptors or receptor MRD status, i.e. disseminated tumor cells (DTCs) or circulating tumor cells (CTCs) as precursors of metastatic lesions [20, 21, 41–43]. The presence of CTCs in peripheral blood is a known independent predictor of poor progression free survival (PFS) and overall survival (OS) in MBC [20, 44–47].

Further clinical studies are required to analyze the potential benefit of extending the indication for HER2-targeted therapies, for example, for brain-metastasized patients with initially triple-negative BC PTs, either via dual blockade by trastuzumab/pertuzumab or via agents such as T-DM1, which was shown to be superior in the EMILIA study [48, 49], or possibly lapatinib, a dual tyrosine kinase inhibitor that interferes with both the epidermal growth factor receptor (EGFR) and HER2 signaling pathways. A recently developed murine model suggests improved anti-tumor efficacy with lapatinib-loaded human serum albumin nanoparticles in triple-negative BC metastasis to the brain [50].

Determination of HER2 status of CTCs may help to optimize individualized treatment solutions, especially in the case of metastatic sites which are unavailable or suboptimal for rebiopsy. Moreover, HER2-CTC positivity in patients with initially triple-negative PT could serve as an additional criterion to perform cerebral imaging earlier, before the onset of severe neurological symptoms. In this context, the DETECT Study group has addressed the value of CTC HER2 overexpression in predicting the HER2 status of metastases. However, they were unable to demonstrate any significant change in HER2 expression between PT and MT [51].

### Concordance of RT-qPCR and IHC in assessing tumor biomarkers in the PT and MT settings

In our study, assessments of BC biomarkers and their dynamics from PT to MT showed a high degree of concordance between mRNA quantification with the well-established IHC methodology, the gold standard in immunophenotyping.

As shown in Table 2, the concordance of these two methodologies was 81% (κ 0.51) in PT vs. 84% (κ 0.34) in MT for ER/*ESR1*; 70% (κ 0.10) in PT vs. 78% (κ −0.32) in MT for PR/*PGR*; and 100% in PT vs. 89% in MT for HER2/*ERBB2*. These concordance rates are consistent with multiple recent prospective-retrospective studies using a similar mRNA quantification methodology. Both the FinHer and the S080 trial reported concordance rates > 90% between RT-qPCR and image-based IHC [52].

Several methodological limitations to measuring biomarker changes as reported here warrant discussion. On the one hand, the feasibility of rebiopsies, especially before each new therapy line, is limited at various metastatic sites. On the other hand, tumor heterogeneity both within the PT and the MT sites [40] as well as between these two entities and the surrogate markers of MRD, foremost the CTCs, present further limitations.

Another limitation to our findings arises from the structure of our patient population, which was a series of cases rather than a representative general population sample. The relatively young age at primary diagnosis can be explained by the large number of triple-negative cases (25%) and brain metastases (28%).

## CONCLUSIONS

We observed a tendency in biomarker conversion towards a more aggressive phenotype with significant downregulation of *ESR1* and *PGR* in biopsies from all MT sites and in all molecular subtypes of MT, as well as a boost in the proliferation marker *MKI67* in all phenotypes (*p <* 0.001) The discordance in mRNA biomarker assessment between PT and MT due to receptor conversion necessitates dynamic monitoring of tumor biomarkers, possibly via liquid biopsy, e.g. circulating tumor cells (CTCs), or rebiopsy of metastatic lesions.

In patients with initially triple-negative PTs, detection of increased HER2 levels in the MT biopsy correlates significantly with brain and bone metastatic progression. To the best of our knowledge, this study is one of the first so far to imply the correlation of HER2 overexpression in initially triple-negative BC and brain metastasis. This tendency could be caused by substantial HER2/*ERBB2* upregulation or clonal progression in the course of the BC metastatic process. In this context, robust real-time HER2 monitoring of CTC-HER2 may, hypothetically, predict metastasis to brain or bone and affect diagnostic and therapeutic decision making. Determining the HER2 status of CTCs might help to optimize individualized treatment solutions, especially in the case of metastatic sites which are unavailable or suboptimal for rebiopsy.

Overall, RT-qPCR assessment of breast cancer target genes and their mRNA expression is methodologically highly concordant with IHC protein analysis of the tumor biology of both PTs and MTs, further supporting multiple recent prospective-retrospective studies based on similar mRNA quantification methodology. The hypothesis-generating nature of the findings from the present study calls for additional studies in order to comprehensively establish and broaden the evidence base for this technique.

## MATERIALS AND METHODS

### Patients

The patient cohort was selected by screening the tumor banks of two institutions (Heidelberg University Hospital, Heidelberg, Germany and Zurich University Hospital, Zurich, Switzerland) for formalin-fixed, paraffin-embedded (FFPE) matched pairs of primary and metastatic BC tissue samples.

Patients were enrolled in the study from April 2011 through May 2015. The inclusion criterion was enrollment in MBC studies in Heidelberg or Zurich with the availability of matched pair samples of the primary breast tumor and an appropriate metastatic biopsy, irrespective of age at initial diagnosis or metastatic entity.

This study was a part of a previously approved breast cancer project at the University of Zurich, ethics approval no. KEK-2012-553. Ethical approval for the cohort from Heidelberg was obtained from the Ethics Committee of the Medical Faculty of the University of Heidelberg, approval no. S-295/2009.

### Immunohistochemistry (IHC)

Conventional immunostaining of sections of FFPE primary and metastatic tumor tissue for ER, PR, HER2, and Ki-67 was performed at the pathology laboratories of the two study sites according to their local standard procedures for ER (clone 1D5), PR (clone PgR636), and HER2 (A0485) in both PT and MT tissue.

ER and PR results were considered positive if at least 10% of cancer cells stained positive. This cut-off was chosen for two reasons: (1) to be able to distinguish more clearly between hormone-receptor positivity and negativity and (2) because low ER+/HER2− tumors have been shown to be more similar to triple-negative tumors than the usual type of ER+ tumors, and are not clearly endocrine responsive [53].

Ki-67 assays were analyzed by determining the proportion of positively stained relative to all cancer cell nuclei in the tissue section, expressed as a percentage between 0 and 100%. Pathologists at the respective institution interpreted the ER, PR, HER2, and Ki-67 immunostaining results in accordance with local standard practice.

### Chromogenic *in situ* hybridization (CISH)

Tumors with a score of 2+ or 3+ (on a scale from 0 to 3+) for HER2 expression as determined by IHC were further analyzed for *HER2* gene amplification by CISH in one of two central laboratories. The *HER2* status was considered positive if six or more gene copies per nucleus were present. HER2 status was considered positive if CISH for *HER2* was positive, and negative if CISH was negative, regardless of the degree of HER2 protein expression as determined by IHC [52].

### Real time qPCR (RT-qPCR)

RNA was extracted using a bead-based extraction method (RNXtract^®^ IVD kits, BioNTech Diagnostics GmbH, Mainz, Germany). Multiplex RT-qPCR utilized MammaTyper IVD^®^ kits for *ESR1/PGR/ERBB2* and *MKI67* (BioNTech Diagnostics GmbH). After pathology confirmed that tissue sections were representative for the presence of cancer, a single whole-face 10-μm-thick slice from each FFPE tumor block was processed with the RNXtract^®^ RNA extraction kit (BioNTech Diagnostics GmbH) using a magnetic particle-based assay, according to previously established protocols [52]. RT-qPCR was performed using the MammaTyper^®^ kit (BioNTech Diagnostics GmbH) for *ESR1, PGR, ERBB2*, and *MKI67*, and the two reference genes B2M and CALM2, on a Versant® kPCR system (Siemens, Erlangen, Germany). This involved applying one cycle of primer-specific reverse transcription followed by 40 cycles of nucleic acid amplification [52, 54]. The median quantification cycles (Cq) for each of the four genes of interest (GOI) were normalized against the two reference genes (REF) and presented as ΔΔCq values relative to the positive control, obtained after subtracting the ΔCq value of the positive control (pc) from the ΔCq of the sample (s) by the formula: 40−ΔΔCq(GOI)_s_ = 40 − ((Cq[GOI]_s_ − mean Cq[REF]_s_) − (Cq[GOI]_pc_ − mean Cq[REF]_pc_)).

Sensitivity studies as previously reported [55] were performed to exclude the major influence of varying tumor cell content for assay results. A number of cases with particularly low levels of invasive carcinoma and varying amounts of ductal carcinoma *in situ* were analyzed before and after macrodissection, subsequently confirming that tumor cell content did not influence the final test result. This enabled the exclusion of any major influence of tumor cell content (TCC) on *ERBB2, ESR1, PGR*, and *MKI67* mRNA expression.

### Definition of breast cancer biological subtypes

After identifying each of the four biomarkers as positive or negative, the molecular subtype of each tumor was determined using the currently proposed IHC-based breast cancer molecular subtyping algorithm [56]. In concordance with previous studies using mRNA phenotype assessment [52], luminal A cancers were defined as having a high *ESR1* or *PGR* mRNA content and a low *ERBB2* and *MKI67* content. Luminal B cancers were defined as having high cancer and *MKI67* content, or high *ESR1* content but low *PGR* and *ERBB2* content. Triple-negative cancers comprised those cancers that had low *ESR1, PGR* and *ERBB2* mRNA content, irrespective of cancer *MKI67* mRNA content.

The same scheme was used to categorize the cancers according to the IHC and CISH results, but using protein expression (at IHC) and the number of HER2 gene copies (at CISH) instead of cancer mRNA content. For example, cancers that were ER and PR positive (with ≥10% of nuclei positive in each staining) and HER2 negative (by CISH), and had low Ki-67 (<20% of nuclei staining positive at IHC) were considered luminal A cancers. This cut-off was chosen based on our own cases and Ki-67 staining results [57] as well as the cut-off from the 2013 St. Gallen consensus [56].

### Statistical methods

To analyze and compare quantitative RT-qPCR marker levels, this study explored the changes during tumor progression (by subtype), location, and time interval. RT-qPCR and IHC results were compared between paired primary and metastatic sites. RNA levels were normalized according to the 40−ΔΔCq method. Changes in RNA marker levels were analyzed qualitatively and quantitatively to visualize specific biomarker profiles based on tumor subtype, metastatic site, and time between PT and MT biopsy.

Demographic data and clinical characteristics were described as frequency and percentage, median and range, or mean and standard deviation. Groups were compared using the Wilcoxon/Mann–Whitney rank test or Fisher's exact test, as appropriate. A significance level of 5% was chosen.

## Abbreviations

BC: breast cancer
MBC: metastatic breast cancer
PT: primary tumor
MT: metastatic tumor
DTCs: disseminated tumor cells
CTCs: circulating tumor cells
ER: estrogen receptor
PR: progesterone receptor
HER2: human epidermal growth factor receptor 2
CISH: Chromogenic *in situ* hybridization
*ESR1*: Estrogen Receptor 1 gene
*PGR*: Progesterone Receptor gene
*ERBB2*: Receptor tyrosine-protein kinase erbB-2 gene
IHC: immunohistochemistry
RT-qPCR: real-time quantitative polymerase chain reaction
EGFR: epidermal growth factor receptor
TCC: tumor cell content
MRD: minimal residual disease.
